# The genome sequence of the Birch Marble, *Apotomis betuletana* (Haworth, 1811)

**DOI:** 10.12688/wellcomeopenres.18984.1

**Published:** 2023-02-09

**Authors:** Douglas Boyes, University of Oxford and Wytham Woods Genome Acquisition Lab, Darwin Tree of Life Barcoding collective, Wellcome Sanger Institute Tree of Life programme, Wellcome Sanger Institute Scientific Operations: DNA Pipelines collective, Tree of Life Core Informatics collective, John F. Mulley, Darwin Tree of Life Consortium

**Affiliations:** 1 UK Centre for Ecology and Hydrology, Wallingford, Oxfordshire, UK; 2 School of Natural Sciences, Bangor University, Bangor, Wales, UK

**Keywords:** Apotomis betuletana, Birch Marble, genome sequence, chromosomal, Lepidoptera

## Abstract

We present a genome assembly from an individual male *Apotomis betuletana* (the Birch Marble; Arthropoda; Insecta; Lepidoptera; Tortricidae). The genome sequence is 684 megabases in span. Most of the assembly is scaffolded into 28 chromosomal pseudomolecules with the Z sex chromosome assembled. The mitochondrial genome has also been assembled and is 15.8 kilobases in length. Gene annotation of this assembly on Ensembl identified 21,717 protein coding genes.

## Species taxonomy

Eukaryota; Metazoa; Ecdysozoa; Arthropoda; Hexapoda; Insecta; Pterygota; Neoptera; Endopterygota; Lepidoptera; Glossata; Ditrysia; Tortricoidea; Tortricidae; Olethreutinae; Olethreutini; *Apotomis*; *Apotomis betuletana* ( Haworth, 1811) (NCBI:txid1100915).

## Background

The birch marble *Apotomis betuletana* (Tortricidae: Olethreutinae) is a relatively large (16–20 mm wingspan) ‘micromoth’, widely reported from across the Western palearctic. *A. betuletana* was classified as ‘common’ in a recent assessment of British microlepidoptera ( Davis, 2012). Adults fly between June and September in the UK and are readily attracted to light, and traps baited with (Z)-10-tetradecenyl acetate will attract males ( Booij & Voerman, 1984). The species was named in 1811 as *Tortrix betuletana* by Adrian Hardy Haworth in volume III of his ‘Lepidoptera Britannica’ ( Haworth, 1811), with the species name recognising the association with birch ( *Betula* sp.), which is the primary, and possibly only, larval foodplant. Larvae feed in rolled and folded birch leaves during early summer.

The basal two-thirds of the adult wing are dark brown to black, with the distal third white, sometimes with small yellowish or brown spots or patches. The overall appearance, as with several other genera of Tortricidae, is of a bird dropping, and the species is often referred to as a bird dropping mimic. Cott, in his 1940 work on adaptive colouration, considered this phenomenon to be a case of ‘special resemblance’ ( Cott, 1940), but it is actually an example of masquerade. Masquerade is differentiated from crypsis in that the latter makes potential prey items difficult to detect and therefore confuses predators’ sensory processes, whereas masquerading individuals resemble inanimate, typically inedible, items, such as sticks, stones, or bird droppings, which confuse predators’ cognitive abilities ( Skelhorn *et al.*, 2010). Masquerade is most effective when the object being copied is common in the environment ( Skelhorn *et al.*, 2011; Skelhorn, 2015), raising the possibility that declines in woodland birds ( Burns *et al.*, 2020) may counterintuitively lead to a decline in the abundance of this species.

The genome of *A. betuletana* will aid research into mechanisms of masquerade in Lepidoptera. Here we present a chromosomally complete genome sequence for *A. betuletana*, based on one male specimen from Wytham Woods, Oxfordshire.

## Genome sequence report

**Figure 1.**
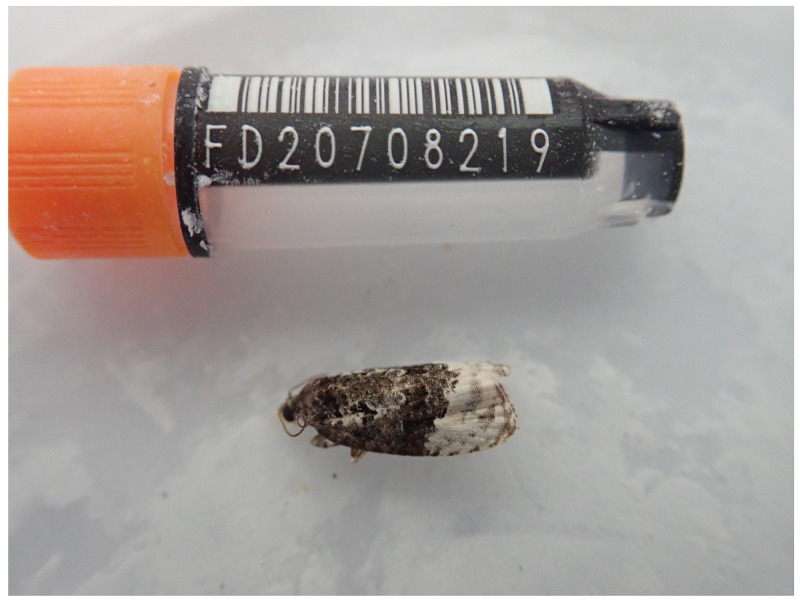
Image of the *Apotomis betuletana* (ilApoBetu1) specimen used for genome sequencing.

The genome was sequenced from one male *Apotomis betuletana* ( Figure 1) collected from Wytham Woods, UK (latitude 51.77, longitude –1.33). A total of 34-fold coverage in Pacific Biosciences single-molecule HiFi long reads and 71-fold coverage in 10X Genomics read clouds were generated. Primary assembly contigs were scaffolded with chromosome conformation Hi-C data. Manual assembly curation corrected 27 missing or mis-joins and removed seven haplotypic duplications, reducing the scaffold number by 24.05%.

**Figure 2.**
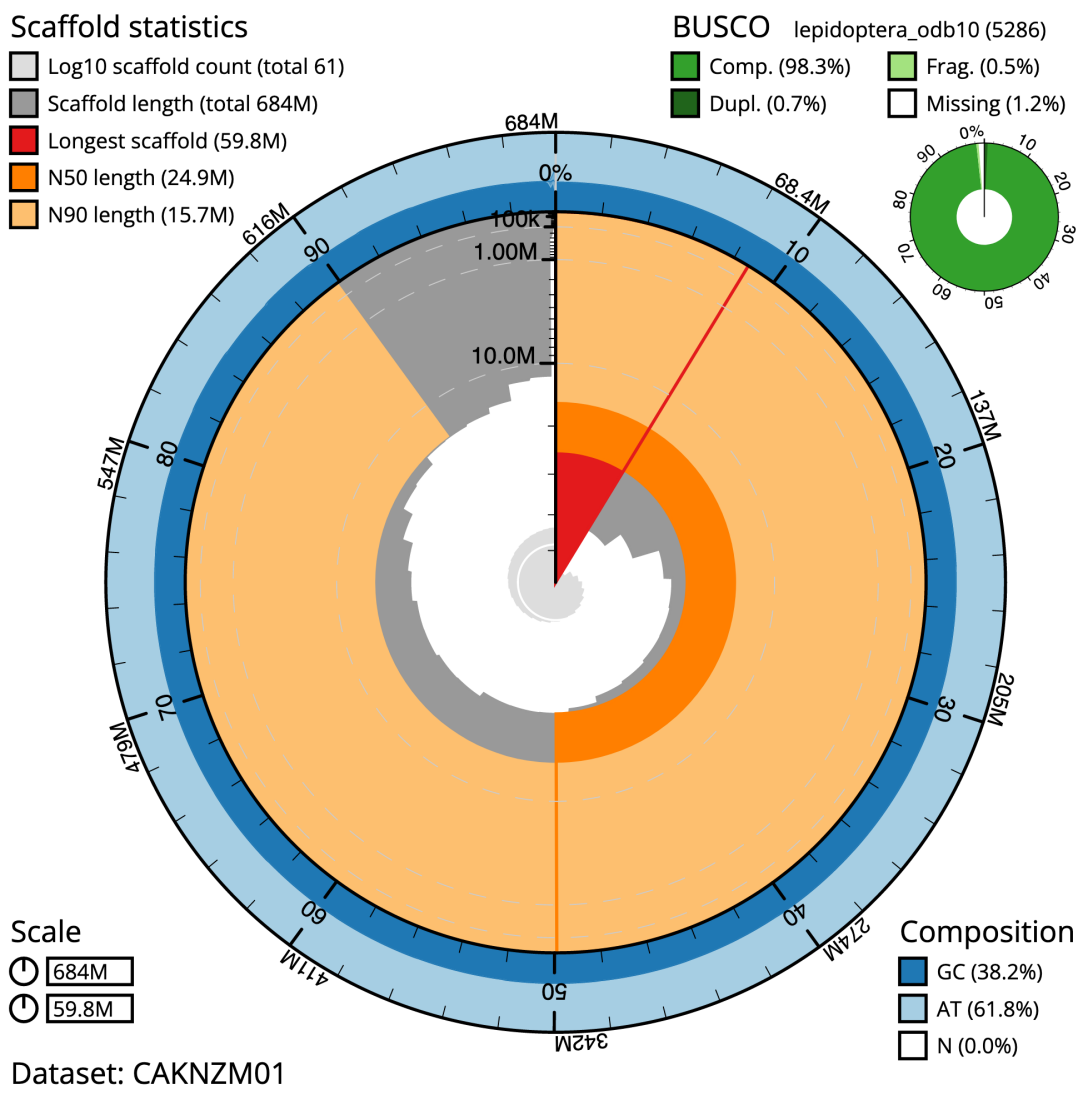
Genome assembly of *Apotomis betuletana*, ilApoBetu1.1: metrics. The BlobToolKit Snailplot shows N50 metrics and BUSCO gene completeness. The main plot is divided into 1,000 size-ordered bins around the circumference with each bin representing 0.1% of the 684,240,599 bp assembly. The distribution of scaffold lengths is shown in dark grey with the plot radius scaled to the longest scaffold present in the assembly (59,814,915 bp, shown in red). Orange and pale-orange arcs show the N50 and N90 scaffold lengths (24,873,308 and 15,741,175 bp), respectively. The pale grey spiral shows the cumulative scaffold count on a log scale with white scale lines showing successive orders of magnitude. The blue and pale-blue area around the outside of the plot shows the distribution of GC, AT and N percentages in the same bins as the inner plot. A summary of complete, fragmented, duplicated and missing BUSCO genes in the lepidoptera_odb10 set is shown in the top right. An interactive version of this figure is available at https://blobtoolkit.genomehubs.org/view/ilApoBetu1.1/dataset/CAKNZM01/snail.

The final assembly has a total length of 684.2 Mb in 60 sequence scaffolds with a scaffold N50 of 24.9 Mb ( Table 1). Most (99.63%) of the assembly sequence was assigned to 28 chromosomal-level scaffolds, representing 27 autosomes and the Z sex chromosome. Chromosome-scale scaffolds confirmed by the Hi-C data are named in order of size ( Figure 2– Figure 5; Table 2). The assembly has a BUSCO v5.3.2 ( Manni *et al.*, 2021) completeness of 98.3% (single 97.5%, duplicated 0.7%) using the lepidoptera_odb10 reference set. While not fully phased, the assembly deposited is of one haplotype. Contigs corresponding to the second haplotype have also been deposited.

**Table 1.** Genome data for *Apotomis betuletana*, ilApoBetu1.1.

Project accession data		
Assembly identifier	ilApoBetu1.1	
Species	*Apotomis betuletana*	
Specimen	ilApoBetu1	
NCBI taxonomy ID	1100915	
BioProject	PRJEB49039	
BioSample ID	SAMEA7701588	
Isolate information	ilApoBetu1 (PacBio and 10X) ilApoBetu2 (Hi-C)	
Assembly metrics *		*Benchmark*
Consensus quality (QV)	57.6	*≥ 50*
*k*-mer completeness	99.99%	*≥ 95%*
BUSCO **	C:98.3%[S:97.5%,D:0.7%], F:0.5%,M:1.2%,n:5,286	*C ≥ 95%*
Percentage of assembly mapped to chromosomes	99.63%	*≥ 95%*
Sex chromosomes	Z chromosome	*localised homologous pairs*
Organelles	Mitochondrial genome assembled	*complete single alleles*
Raw data accessions		
PacificBiosciences SEQUEL II	ERR7815860	
10X Genomics Illumina	ERR7440907–ERR7440910	
Hi-C Illumina	ERR7569936	
Genome assembly		
Assembly accession	GCA_932273695.1	
*Accession of alternate haplotype*	GCA_932273875.1	
Span (Mb)	684.2	
Number of contigs	134	
Contig N50 length (Mb)	12.4	
Number of scaffolds	60	
Scaffold N50 length (Mb)	24.9	
Longest scaffold (Mb)	59.8	
Genome annotation		
Number of protein-coding genes	21,717	
Number of gene transcripts	21,968	

* Assembly metric benchmarks are adapted from column VGP-2020 of “Table 1: Proposed standards and metrics for defining genome assembly quality” from ( Rhie *et al.*, 2021). ** BUSCO scores based on the lepidoptera_odb10 BUSCO set using v5.3.2. C = complete [S = single copy, D = duplicated], F = fragmented, M = missing, n = number of orthologues in comparison. A full set of BUSCO scores is available at https://blobtoolkit.genomehubs.org/view/ilApoBetu1.1/dataset/CAKNZM01/busco.

**Figure 3.**
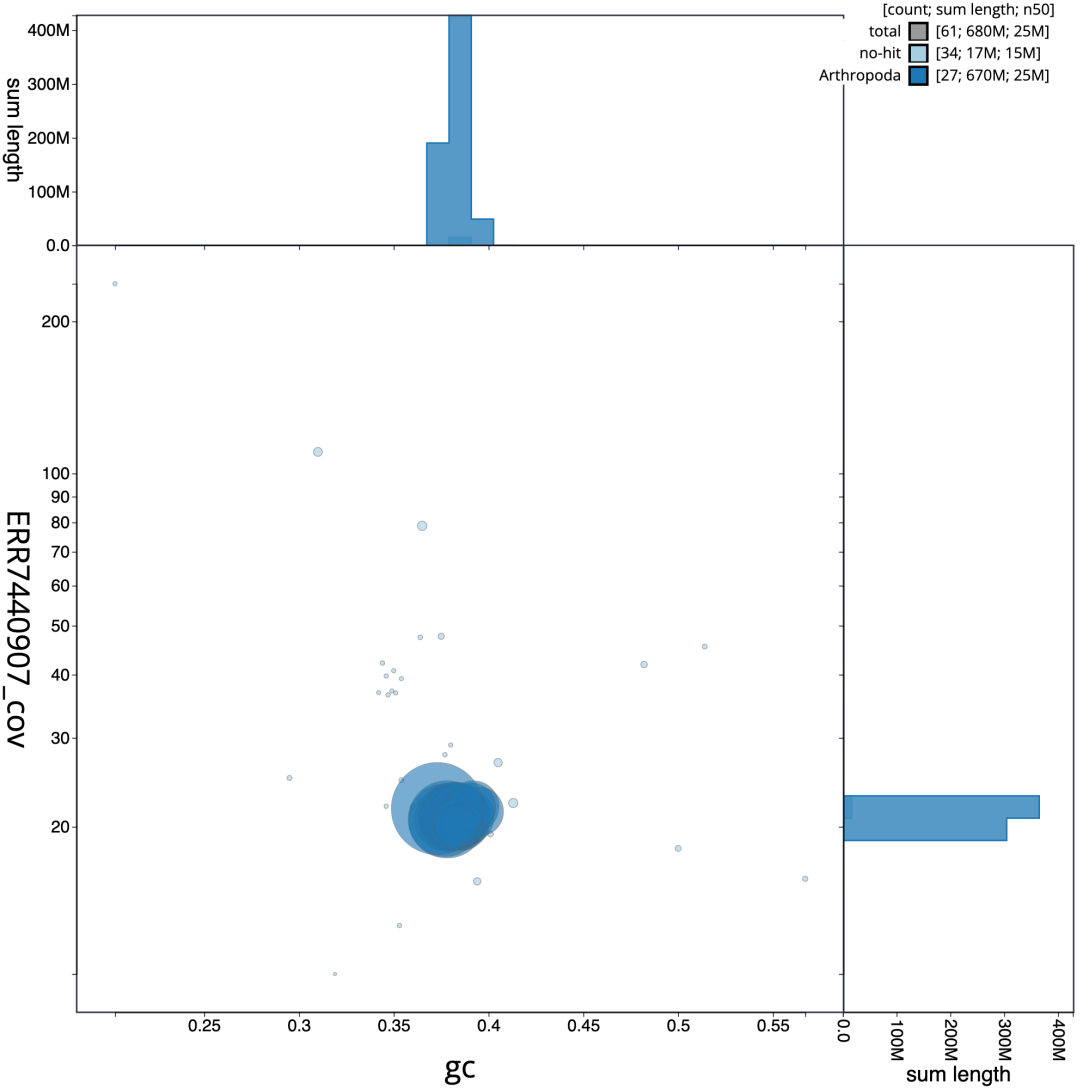
Genome assembly of *Apotomis betuletana*, ilApoBetu1.1: GC coverage. BlobToolKit GC-coverage plot. Scaffolds are coloured by phylum. Circles are sized in proportion to scaffold length. Histograms show the distribution of scaffold length sum along each axis. An interactive version of this figure is available at https://blobtoolkit.genomehubs.org/view/ilApoBetu1.1/dataset/CAKNZM01/blob.

**Figure 4.**
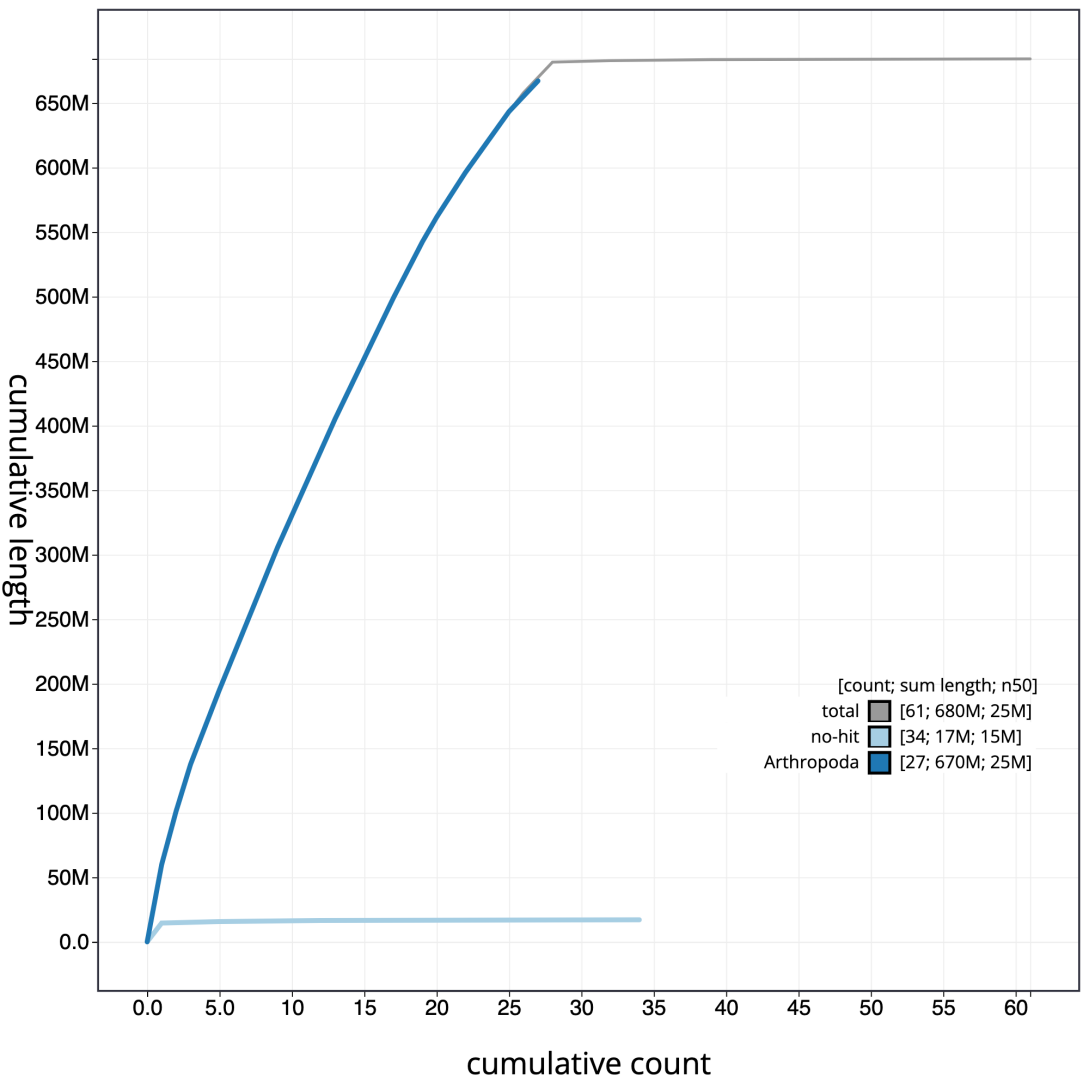
Genome assembly of *Apotomis betuletana*, ilApoBetu1.1: cumulative sequence. BlobToolKit cumulative sequence plot. The grey line shows cumulative length for all scaffolds. Coloured lines show cumulative lengths of scaffolds assigned to each phylum using the buscogenes taxrule. An interactive version of this figure is available at https://blobtoolkit.genomehubs.org/view/ilApoBetu1.1/dataset/CAKNZM01/cumulative.

**Figure 5.**
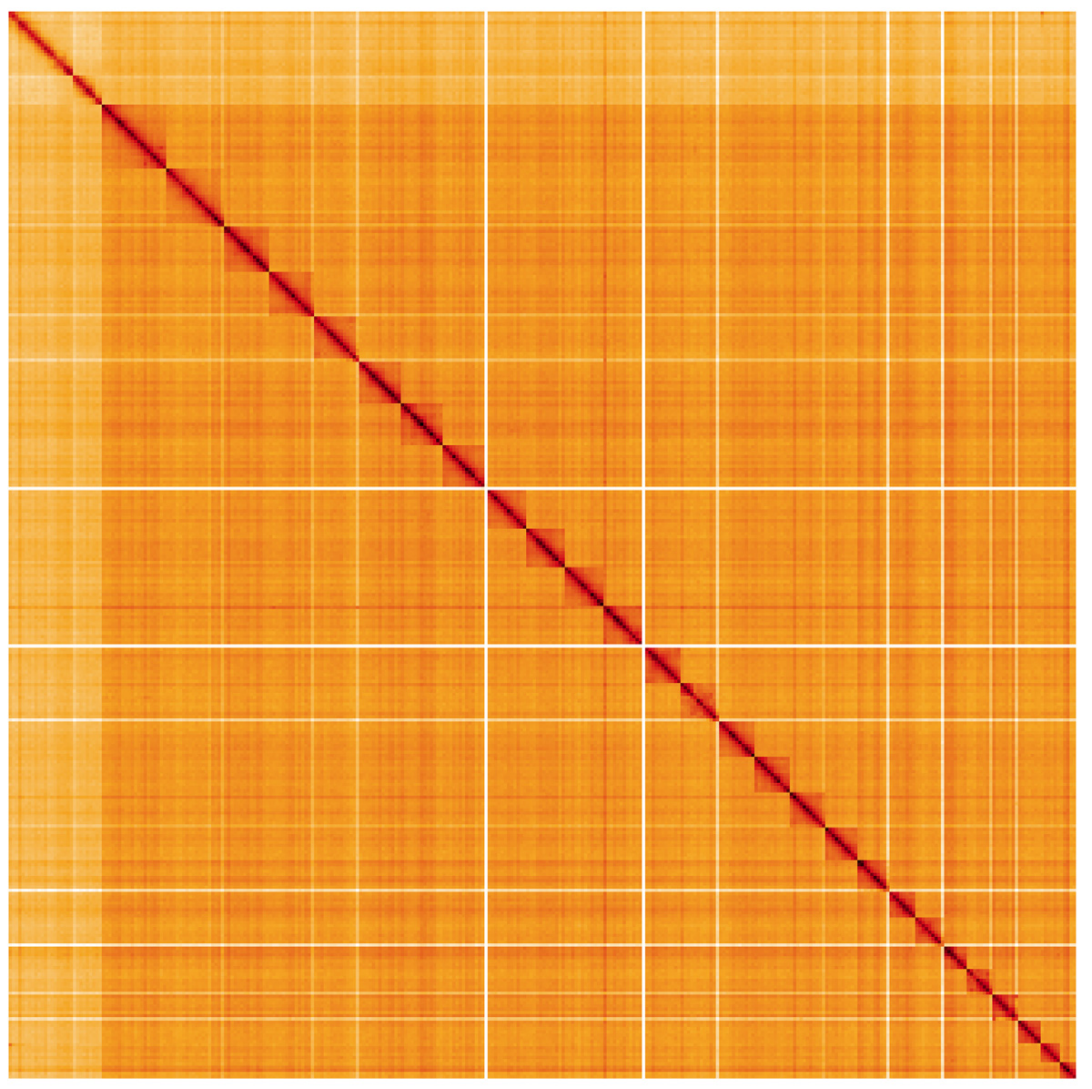
Genome assembly of *Apotomis betuletana*, ilApoBetu1.1: Hi-C contact map. Hi-C contact map of the ilApoBetu1.1 assembly, visualised using HiGlass. Chromosomes are shown in order of size from left to right and top to bottom. An interactive version of this figure may be viewed at https://genome-note-higlass.tol.sanger.ac.uk/l/?d=DsBR-8qXSpyKsith2rNGKw.

**Table 2.** Chromosomal pseudomolecules in the genome assembly of *Apotomis betuletana*, ilApoBetu1.

INSDC accession	Chromosome	Size (Mb)	GC%
OW026299.1	1	41.42	37.8
OW026300.1	2	36.39	37.7
OW026301.1	3	29.81	38.1
OW026302.1	4	28.07	37.8
OW026303.1	5	27.84	38.1
OW026304.1	6	27.76	38.4
OW026305.1	7	27.36	38.3
OW026306.1	8	26.96	38
OW026307.1	9	25.71	38.2
OW026308.1	10	24.87	38.4
OW026309.1	11	24.76	38.2
OW026310.1	12	24.8	37.9
OW026311.1	13	23.63	38
OW026312.1	14	23.53	38.5
OW026313.1	15	23.02	38.3
OW026314.1	16	22.98	38.4
OW026315.1	17	22.07	38.5
OW026316.1	18	21.35	38.8
OW026317.1	19	19.4	38.5
OW026318.1	20	17.54	39.2
OW026319.1	21	17.27	38.4
OW026320.1	22	15.82	39.5
OW026321.1	23	15.74	38.4
OW026322.1	24	15.48	39.1
OW026323.1	25	14.55	38.5
OW026324.1	26	12.06	38.6
OW026325.1	27	11.69	38.3
OW026298.1	Z	59.81	37.3
OW026326.1	MT	0.02	20.3
-	unplaced	2.52	38.6

## Genome annotation report

The *A. betuletana* genome assembly GCA_932273695.1 was annotated using the Ensembl rapid annotation pipeline ( Table 1; https://rapid.ensembl.org/Apotomis_betuletana_GCA_932273695.1/). The resulting annotation includes 21,968 transcribed mRNAs from 21,717 protein-coding genes.

## Methods

### Sample acquisition and nucleic acid extraction

Two *A. betuletana* specimens (ilApoBetu1 and ilApoBetu2) were collected in Wytham Woods, Oxfordshire (biological vice-county: Berkshire), UK (latitude 51.77, longitude –1.33) on 31 August 2020 by netting. The specimens were collected and identified by Douglas Boyes (University of Oxford) and snap-frozen on dry ice.

DNA was extracted at the Tree of Life laboratory, Wellcome Sanger Institute (WSI). The ilApoBetu1 sample was weighed and dissected on dry ice. Whole organism tissue was *d*isrupted using a Nippi Powermasher fitted with a BioMasher pestle. High molecular weight (HMW) DNA was extracted using the Qiagen MagAttract HMW DNA extraction kit. Low molecular weight DNA was removed from a 20 ng aliquot of extracted DNA using 0.8X AMpure XP purification kit prior to 10X Chromium sequencing; a minimum of 50 ng DNA was submitted for 10X sequencing. HMW DNA was sheared into an average fragment size of 12–20 kb in a Megaruptor 3 system with speed setting 30. Sheared DNA was purified by solid-phase reversible immobilisation using AMPure PB beads with a 1.8X ratio of beads to sample to remove the shorter fragments and concentrate the DNA sample. The concentration of the sheared and purified DNA was assessed using a Nanodrop spectrophotometer and Qubit Fluorometer and Qubit dsDNA High Sensitivity Assay kit. Fragment size distribution was evaluated by running the sample on the FemtoPulse system.

### Sequencing

Pacific Biosciences HiFi circular consensus and 10X Genomics read cloud DNA sequencing libraries were constructed according to the manufacturers’ instructions. DNA sequencing was performed by the Scientific Operations core at the WSI on Pacific Biosciences SEQUEL II (HiFi) and Illumina NovaSeq 6000 (10X) instruments. Hi-C data were also generated from whole organism tissue of ilApoBetu2 using the Arima v2 kit and sequenced on the Illumina NovaSeq 6000 instrument.

### Genome assembly

Assembly was carried out with Hifiasm ( Cheng *et al.*, 2021) and haplotypic duplication was identified and removed with purge_dups ( Guan *et al.*, 2020). One round of polishing was performed by aligning 10X Genomics read data to the assembly with Long Ranger ALIGN, calling variants with freebayes ( Garrison & Marth, 2012). The assembly was then scaffolded with Hi-C data ( Rao *et al.*, 2014) using YaHS ( Zhou *et al.*, 2022). The assembly was checked for contamination as described previously ( Howe *et al.*, 2021). Manual curation was performed using HiGlass ( Kerpedjiev *et al.*, 2018) and Pretext ( Harry, 2022). The mitochondrial genome was assembled using MitoHiFi ( Uliano-Silva *et al.*, 2022), which performed annotation using MitoFinder ( Allio *et al.*, 2020). The genome was analysed and BUSCO scores were generated within the BlobToolKit environment ( Challis *et al.*, 2020). Table 3 contains a list of all software tool versions used, where appropriate.

**Table 3.** Software tools and versions used.

Software tool	Version	Source
BlobToolKit	3.5.0	Challis *et al.*, 2020
freebayes	1.3.1-17-gaa2ace8	Garrison & Marth, 2012
Hifiasm	0.16.1	Cheng *et al.*, 2021
HiGlass	1.11.6	Kerpedjiev *et al.*, 2018
Long Ranger ALIGN	2.2.2	https://support.10xgenomics.com/genome-exome/ software/pipelines/latest/advanced/other-pipelines
MitoHiFi	2	Uliano-Silva *et al.*, 2022
PretextView	0.2	Harry, 2022
purge_dups	1.2.3	Guan *et al.*, 2020
YaHS	1.0	Zhou *et al.*, 2022

### Genome annotation

The BRAKER2 pipeline ( Brůna *et al.*, 2021) was used in the default protein mode to generate annotation for the *A. betuletana* assembly (GCA_932273695.1) in Ensembl Rapid Release.

### Ethics and compliance issues

The materials that have contributed to this genome note have been supplied by a Darwin Tree of Life Partner. The submission of materials by a Darwin Tree of Life Partner is subject to the Darwin Tree of Life Project Sampling Code of Practice. By agreeing with and signing up to the Sampling Code of Practice, the Darwin Tree of Life Partner agrees they will meet the legal and ethical requirements and standards set out within this document in respect of all samples acquired for, and supplied to, the Darwin Tree of Life Project. All efforts are undertaken to minimise the suffering of animals. Each transfer of samples is further undertaken according to a Research Collaboration Agreement or Material Transfer Agreement entered into by the Darwin Tree of Life Partner, Genome Research Limited (operating as the Wellcome Sanger Institute), and in some circumstances other Darwin Tree of Life collaborators.

## Data Availability

European Nucleotide Archive: *Apotomis betuletana* (birch marble). Accession number PRJEB49039; https://identifiers.org/ena.embl/PRJEB49039. ( Wellcome Sanger Institute, 2021) The genome sequence is released openly for reuse. The *Apotomis betuletana* genome sequencing initiative is part of the Darwin Tree of Life (DToL) project. All raw sequence data and the assembly have been deposited in INSDC databases. Raw data and assembly accession identifiers are reported in Table 1.

## Author information

Members of the University of Oxford and Wytham Woods Genome Acquisition Lab are listed here: https://doi.org/10.5281/zenodo.4789928.

Members of the Darwin Tree of Life Barcoding collective are listed here: https://doi.org/10.5281/zenodo.4893703.

Members of the Wellcome Sanger Institute Tree of Life programme are listed here: https://doi.org/10.5281/zenodo.4783585.

Members of Wellcome Sanger Institute Scientific Operations: DNA Pipelines collective are listed here: https://doi.org/10.5281/zenodo.4790455.

Members of the Tree of Life Core Informatics collective are listed here: https://doi.org/10.5281/zenodo.5013541.

Members of the Darwin Tree of Life Consortium are listed here: https://doi.org/10.5281/zenodo.4783558.
