# Supplementary material for: The genome sequence of the Birch Marble,  Apotomis betuletana (Haworth, 1811)
Source: Wellcome Open Res. 2023 Feb 9;8:66. [Version 1] doi: 10.12688/wellcomeopenres.18984.1 (PMC10111142; doi:10.12688/wellcomeopenres.18984.1)
Supplement: Peer review history

# Peer review: Reviewer response for version 1

**DOI:** 10.21956/wellcomeopenres.21046.r55534

**Published:** 2023-04-17

**Authors:** Mandira Katuwal

**Affiliations:** 1 Department of Molecular and Cellular Biology, Harvard University, Cambridge, Massachusetts, USA

The authors’ effort in sequencing, assembling, and annotating the genome of the birch marble *Apotomis betuletana* using Illumina, PacBio and HI-C reads have resulted in a commendable accomplishment. The chromosome-level annotated genome assembly of the micromoth species is a great addition to the insect genomic resources. The manuscript is well written and presents information in a clear and concise manner. However, some minor comments and suggestions (given below) could improve the manuscript’s clarity and interest to a broad range of readers within the genomics community.

**Broad comments:**

- Some of the references, manufacturer’s information, or catalogue numbers are missing in the manuscript and needs to be added. For example, sequencing instruments and the kits used should be mentioned with the manufacturer's name, country, catalogue number, and the service provider.

- Explaining the rationale of the research project and the future possible application of this genomic resources in a bit more details could provide more insights and make the research more applicable to broad range of readers in the genomics community.

- The manuscript currently only mentions the software used and their versions, but presenting the computational pipeline in the form of a table or a figure would be more informative and reproducible. Additionally, it would be beneficial to deposit any generated codes/scripts in a public repository like Github. It is excellent that the authors have deposited all the sequence data files in the public repository.

**Specific comments**

- Please provide a reference for the statement: “the birch as the primary and possibly only larval food plant”.

- Replace “ *Betula* sp.” by “ *Betula* spp.”

- Please include an explanation for choosing the male moth for sequencing and the reference for Z sex chromosome information.

- Please clarify that the morphology described in the first sentence in the second paragraph under “Background” applies to both males and females or is of the males that were sequenced.

- Please clarify whether the insect gut was included in the “whole organism tissue” for DNA extraction. If it was included, please explain why the insect specimens were dissected prior to DNA sequencing.

- The sentence about extracting high molecular weight DNA using the Qiagen MagAttract HMW DNA extraction kit the “Methods” section is missing the manufacturer or catalogue information, please add this.

- The reference for the Pacific Biosciences HiFi circular consensus and 10X Genomics read cloud DNA sequencing libraries should be included.

- The methodology states that two specimens were used for DNA extraction, but the abstract says that one single male was sequenced. This should be clarified. Also, it is unclear whether ilApoBetu1 was used for both PacBio and 10x libraries.

- In the genome assembly section, please specify what kind of and what percentage contaminants were identified and removed.

- Please add the coverage for Hi-C data.

- Was the repetitive content of the genome analyzed and masked before annotating? Please clarify and add information if it was done. Also, method section says, mito-genome was assembled and annotated provide the details in result section.

Are sufficient details of methods and materials provided to allow replication by others?

Yes

Is the rationale for creating the dataset(s) clearly described?

Yes

Are the datasets clearly presented in a useable and accessible format?

Yes

Are the protocols appropriate and is the work technically sound?

Yes

Reviewer Expertise:

Genomics

I confirm that I have read this submission and believe that I have an appropriate level of expertise to confirm that it is of an acceptable scientific standard.

# Peer review: Reviewer response for version 1

**DOI:** 10.21956/wellcomeopenres.21046.r54808

**Published:** 2023-02-14

**Authors:** Giulio Formenti

**Affiliations:** 1 Vertebrate Genome Laboratory, The Rockefeller University, New York, NY, USA

Boyes et al. report the genome sequence of the Birch Marble, an interesting species from the perspective of studying camouflage. Overall the report is sound. I only have a few minor comments and suggestions below.

- The Pretext image is very pixellated. I know there is a browsable version, but it is not as intuitive. If possible, please increase the resolution of the static image.
- 'With the Z sex chromosome assembled' replace with 'including the Z sex chromosome'
- 'The genome was sequenced from one male Apotomis betuletana' replace with 'The genome was sequenced from one male A. betuletana'
- 'Primary assembly contigs were scaffolded with chromosome conformation Hi-C data - what about the coverage in this case?
- Reducing the scaffold number by 24.05% - how many bases were approximately affected?
- While not fully phased, the assembly deposited is of one haplotype. Contigs corresponding to the second haplotype have also been deposited > it would be more appropriate to talk about haplotigs, since we don't have two haplotypes here (they are mixed)

Are sufficient details of methods and materials provided to allow replication by others?

Yes

Is the rationale for creating the dataset(s) clearly described?

Yes

Are the datasets clearly presented in a useable and accessible format?

Yes

Are the protocols appropriate and is the work technically sound?

Yes

Reviewer Expertise:

genomics

I confirm that I have read this submission and believe that I have an appropriate level of expertise to confirm that it is of an acceptable scientific standard.
